# Supplementary material for: Craniofacial Defects in Embryos with Homozygous Deletion of Eftud2 in Their Neural Crest Cells Are Not Rescued by Trp53 Deletion
Source: Int J Mol Sci. 2022 Aug 12;23(16):9033. doi: 10.3390/ijms23169033 (PMC9409426; doi:10.3390/ijms23169033)
Supplement: Supplementary file 1 [file ijms-23-09033-s001.zip › ijms-1727935-supplementary.pdf]

Table S1. Genotypes of embryos collected from E9.5 to E14.5 (n=9 litters) from mating *Eftud2* $^{+/-}$; *Trp53* $^{loxP/+}$; *Wnt1-Cre2* $^{tg/+}$ with *Eftud2* $^{loxP/loxP}$; *Trp53* $^{loxP/loxP}$, showing that *Eftud2* and *Trp53* genes do not follow Mendelian segregation.

Trp53 $^{loxP/+}$				***Trp53*** $^{loxP/loxP}$				**Total**
		Wnt1-Cre2 $^{tg/+}$				***Wnt1-Cre2*** $^{tg/+}$		
Eftud2 loxP/-	*Eftud2 loxP/+*	*Eftud2 loxP/-*	*Eftud2 loxP/+*	*Eftud2 loxP/-*	*Eftud2 loxP/+*	*Eftud2 loxP/-*	*Eftud2 loxP/+*	
17	3	24	10	5	22	2	10	93

λ^2 P<0.0001

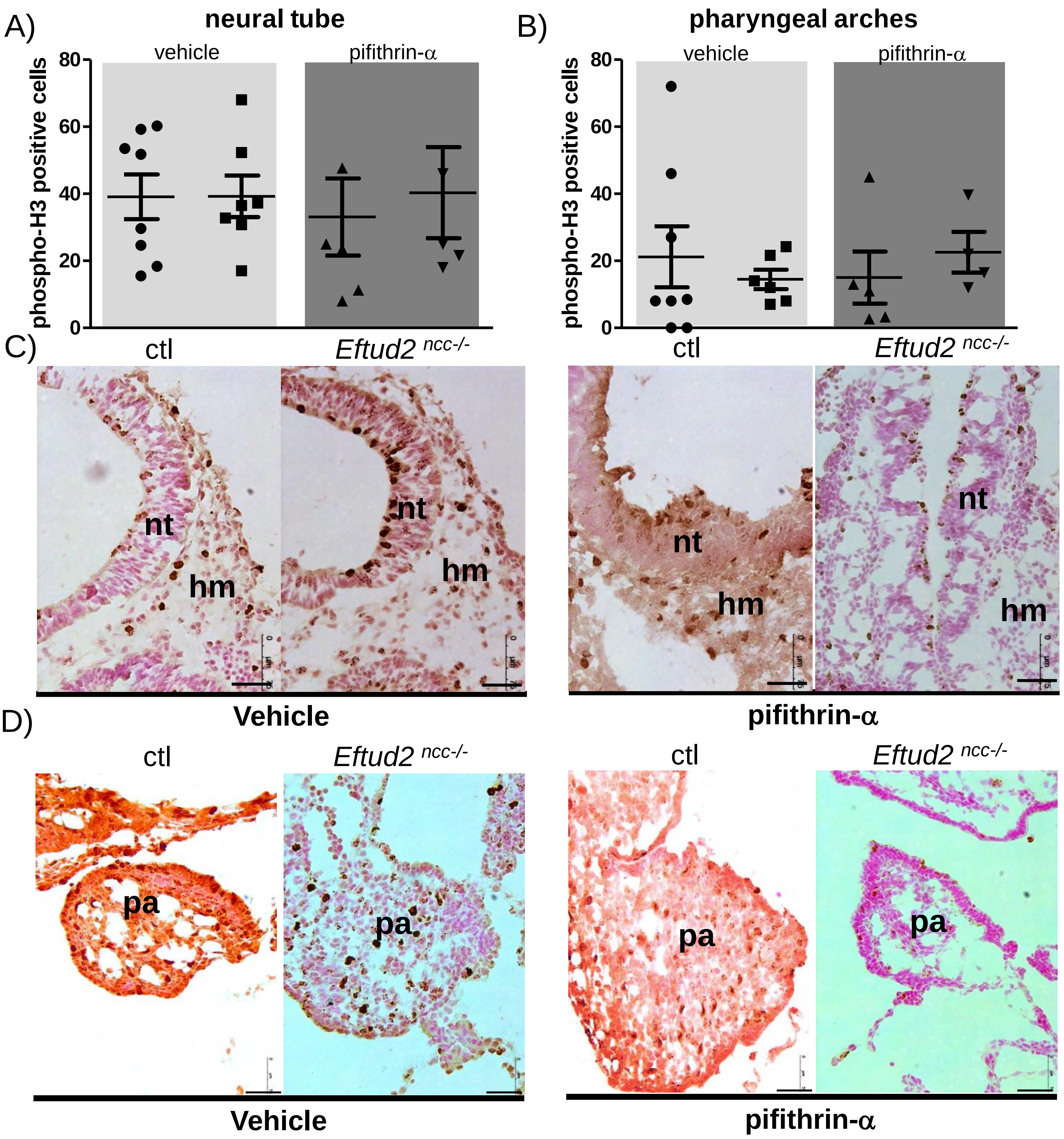

Figure S1. Pifithrin-α did not change proliferation in *Eftud2*$^{ncc-/-}$ mutant E9.5 embryos. (A) Graphs showing no change in the quantification of phospho-H3 positive cells in the neural tube or (B) pharyngeal arches of controls or *Eftud2*$^{ncc-/-}$ mutant E9.5 embryos treated with vehicle (veh) or pifithrin-α (pif). Each dot represents average count in a single embryo. (C) Representative images of phospho-H3 staining by immunohistochemistry of vehicle or pifithrin-α treated E9.5 controls and *Eftud2*$^{ncc-/-}$ mutant embryos. Sections were counterstained with nuclear fast red (red), staining is in brown. nt=neural tube, hm=head mesenchym, pa=pharyngeal arch. Scale bar=50μm.

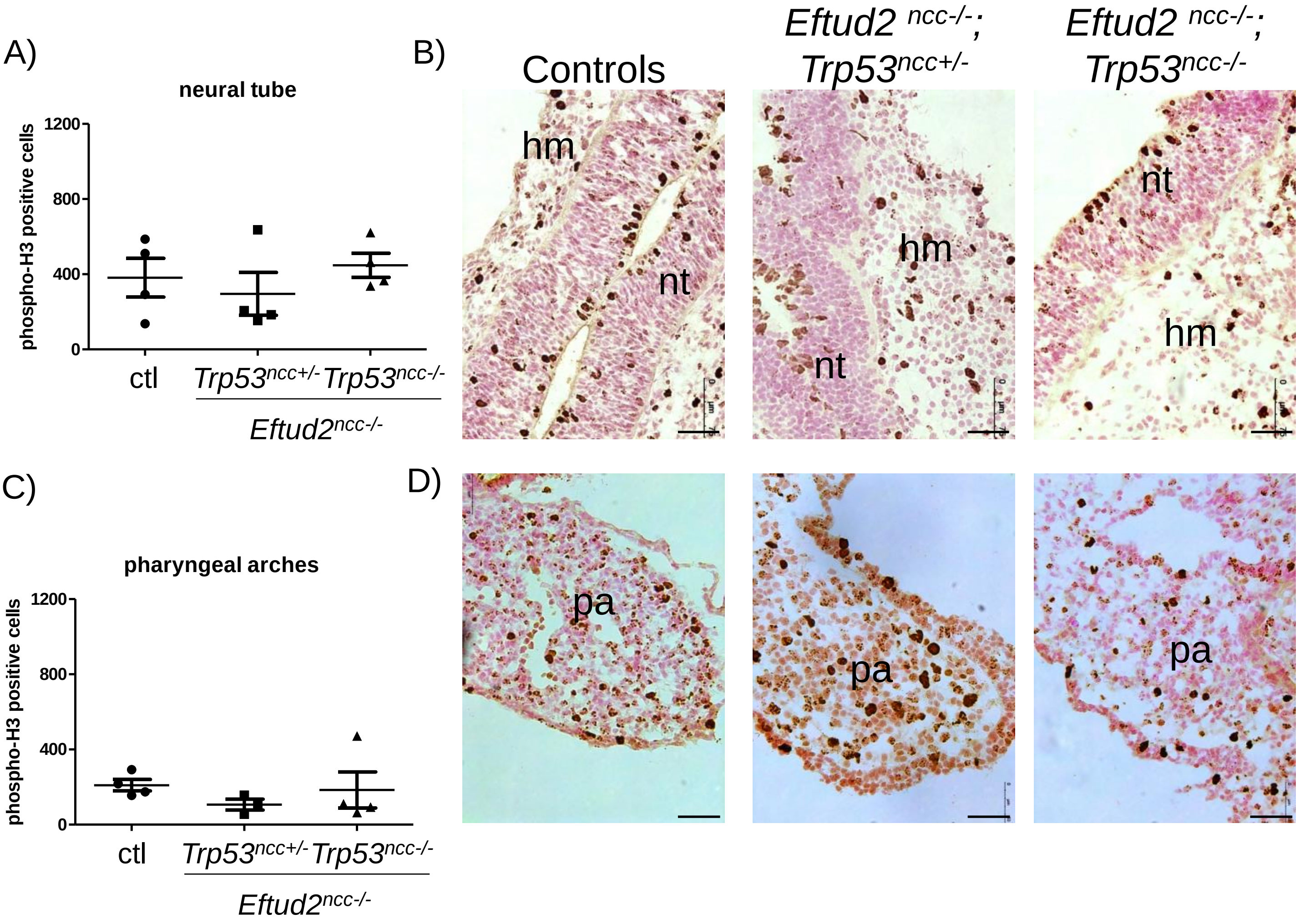

Figure S2. No change in the proliferation levels in E10.5 *Eftud2*$^{ncc-/-}$ mutant with or without *Trp53* compared to controls. (A) Graphs showing no change in the quantification of phospho-H3 positive cells in the neural tube or (C) pharyngeal arches of controls, *Eftud2*$^{ncc-/-}$;*Trp53*$^{ncc+/-}$ and *Eftud2*$^{ncc-/-}$; *Trp53*$^{ncc-/-}$ mutant embryos. Each dot represents average count in a single embryo. (B) Representative images of phospho-H3 staining by immunohistochemistry of controls, *Eftud2*$^{ncc-/-}$;*Trp53*$^{ncc+/-}$ and *Eftud2*$^{ncc-/-}$; *Trp53*$^{ncc-/-}$ mutant E10.5 embryos in the neural tube and (D) pharyngeal arches. Sections were counterstained with nuclear fast red (red), staining is in brown. nt=neural tube, hm=head mesenchym, pa=pharyngeal arch. Scale bar=50µm.

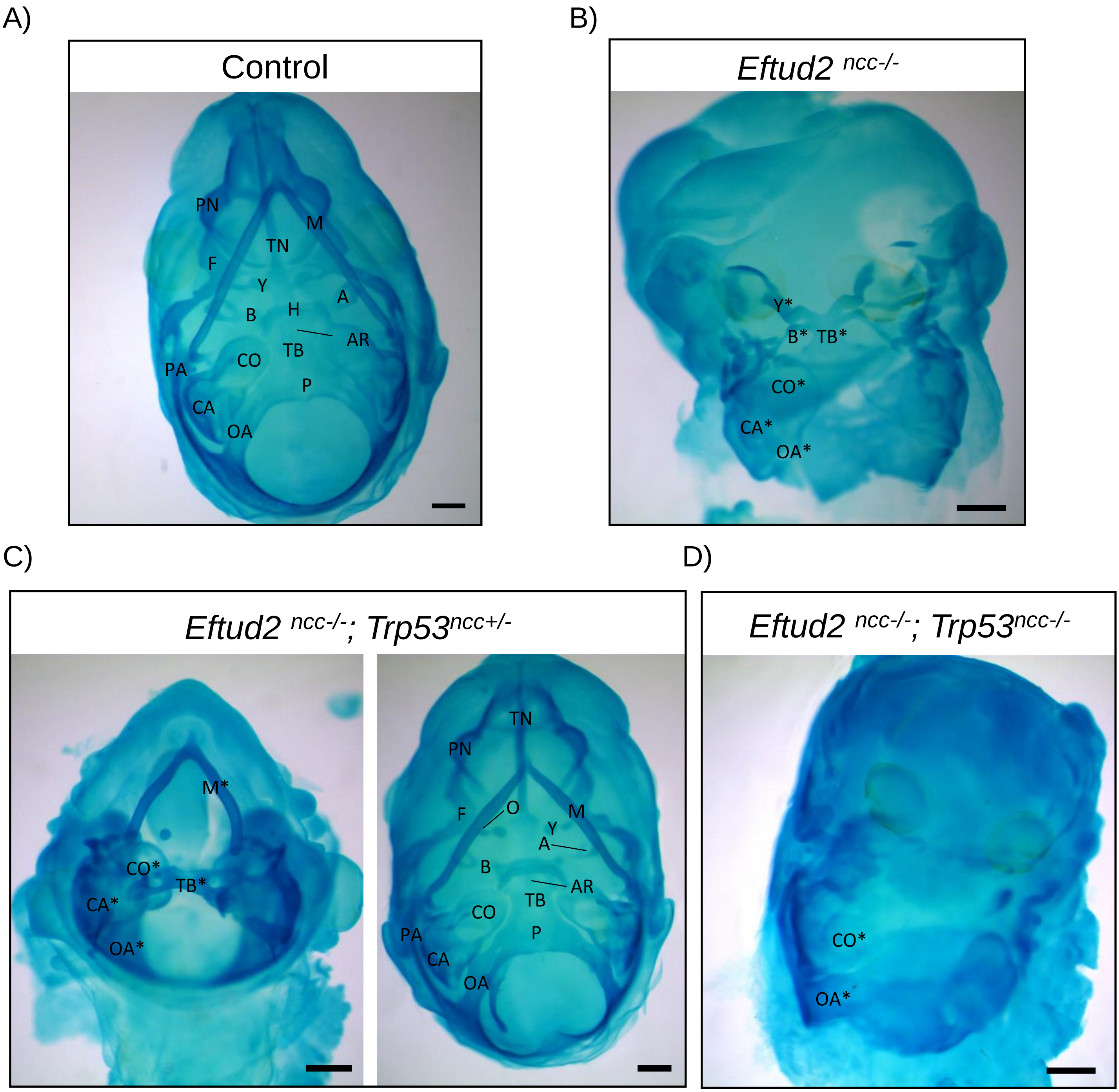

Figure S3. Cartilage formation is abnormal in *Eftud2*$^{ncc-/-}$ mutant E14.5 embryos, regardless of the *Trp53* status. (A) One representative image of a ventral view of Alcian stained control embryo at E14.5 is shown. (B) Representative ventral views of an alive *Eftud2*$^{ncc-/-}$ mutant embryos, as previously described [4]. (C) Representative ventral views of 2/5 alive *Eftud2*$^{ncc-/-}$ *;Trp53*$^{ncc+/-}$ mutant embryos collected showing the range of cartilage abnormalities observed. Among them, one phenotypically normal embryo was collected and is shown in the bottom panel. (D) Ventral view of the single alive *Eftud2*$^{ncc-/-}$*; Trp53*$^{ncc-/-}$mutant embryo collected. Scale bar=500µm. TN=nasal portion of the trabecular plate, PN=paranasal cartilage, F=frontal cartilage, O=orbital cartilage, M=Meckel's cartilage, Y=hypochiasmatic cartilage, B=basitrabecular process, A=ala temporalis cartilage, B=basitrabecular process, AR=acrochordal cartilage, TB= basal portion of the trabecular plate P=parachordal cartilage, OA=occipital arch cartilage, CA=canalicular part of auditory capsule, CO=cochlear part of auditory capsule, PA=parietal cartilage. *Indicates abnormally shaped cartilage.

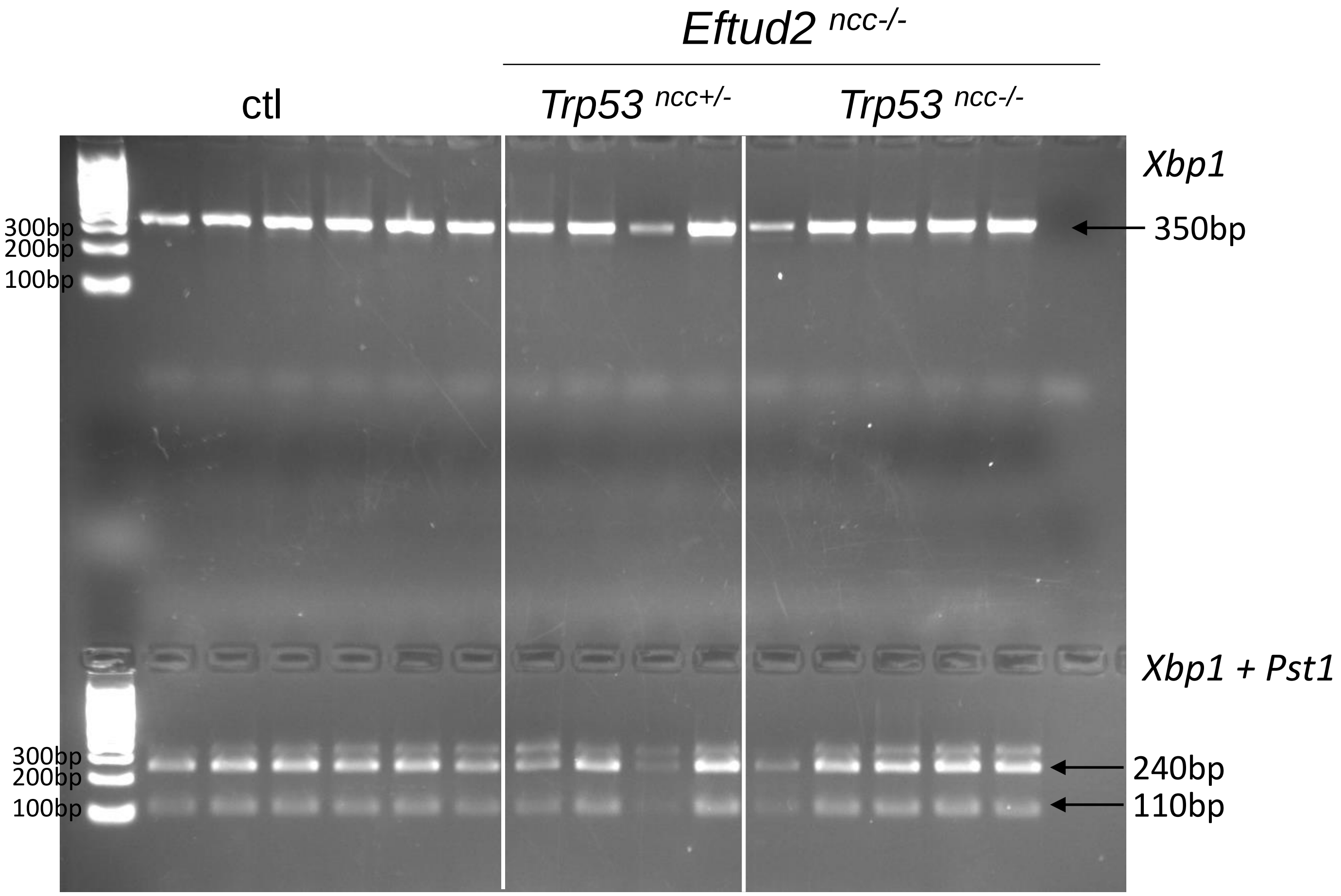

Figure S4. The *Xbp1* splicing arm of the ER-stress pathway is not involved in the craniofacial malformations in *Eftud2*$^{ncc-/-}$ mutants. A cDNA fragment of 350bp of *Xbp1* was amplified by RT-PCR in controls, *Eftud2*$^{ncc-/-}$ *;Trp53*$^{ncc+/-}$ and *Eftud2*$^{ncc-/-}$*; Trp53*$^{ncc-/-}$ mutant E9.5 embryos. Digestion of this cDNA fragment with Pst1 restriction enzyme yields two bands (240 bp and 110 bp) in all of the embryos, indicating an absence of ER stress (see Materials&Methods).

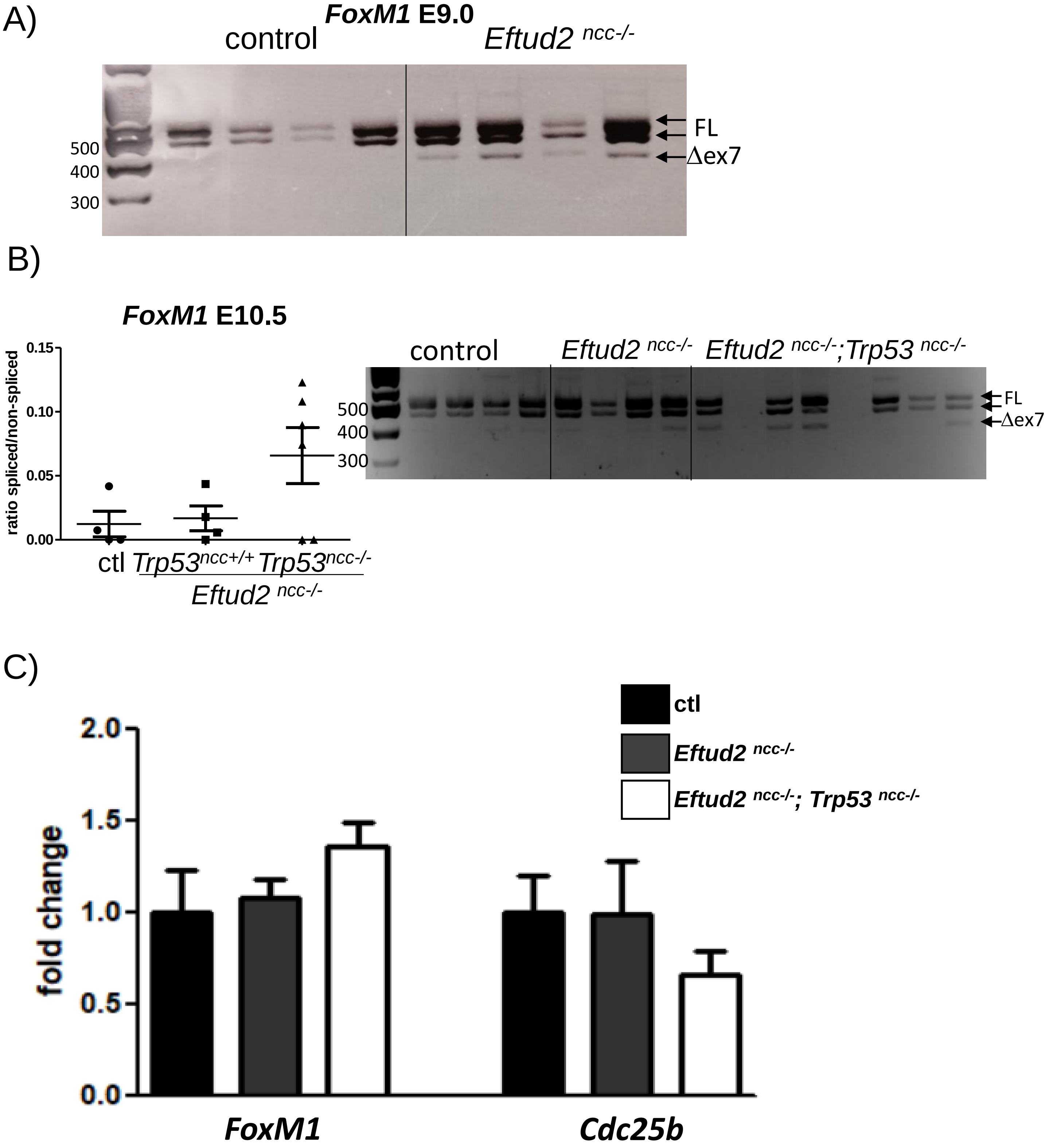

Figure S5. Mis-splicing of *FoxM1* is increased *Eftud2*$^{ncc-/-}$ mutants. (A) Representative gel from RT-PCR analysis showing the presence of a transcript without exon7 of *FoxM1* in *Eftud2*$^{ncc-/-}$ mutant embryos at E9.0, but not in controls. (B) Quantification of the skipped-exon transcript vs the full-length transcript (FL) of *FoxM1* showing a non-significant increase in *Eftud2*$^{ncc-/-}$*; Trp53*$^{ncc-/-}$ mutant E10.5 embryos compared to controls or *Eftud2*$^{ncc-/-}$mutants. A representative gel is on the right. (C) RT-qPCR analysis expressed as fold change over control levels showing no significant change of expression of *FoxM1* and of one of its target *Cdc25b* in controls (n=4), *Eftud2*$^{ncc-/-}$ (n=4) and *Eftud2*$^{ncc-/-}$*; Trp53*$^{ncc-/-}$ (n=6) mutant embryos at E10.5.
